# Examining the gender, ethnicity, and age dimensions of the *healthy immigrant effect*: Factors in the development of equitable health policy

**DOI:** 10.1186/1475-9276-11-8

**Published:** 2012-02-16

**Authors:** Karen M Kobayashi, Steven G Prus

**Affiliations:** 1 Department of Sociology, University of Victoria, PO Box 3050 STN CSC, Victoria, British Columbia, V8W3P5, Canada; 2 Department of Sociology, Carleton University, B750 Loeb Building, 1125 Colonel By Drive, Ottawa, Ontario, K1S5B6, Canada

**Keywords:** *healthy immigrant effect*, gender, ethnicity, mid-life, later life, health care policy

## Abstract

This study expands on previous research on the *healthy immigrant effect *(HIE) in Canada by considering the effects of both immigrant and visible minority status on self-rated health for males and females in mid-(45-64) and later life (65+). The findings reveal a strong HIE among new immigrant middle-aged men, particularly non-Whites. For older men of color the reality is strikingly different: they are disadvantaged in health compared to their Canadian-born counterparts, even when a number of demographic, economic, and lifestyle factors are controlled. Health outcomes for immigrant women are in contrast to that of immigrant men. Among middle-aged women, immigrants, regardless of their ethnicity or number of years since immigration, are much more likely to report poor health compared to the Canadian-born. And, for older women, recent non-white immigrants are more likely to report better health compared to Canadian-born women, although this finding is explained by differences in demographic, economic, and lifestyle factors. Overall, the findings demonstrate the importance of considering the intersections of age, gender, and ethnicity for policymakers in assessing the health of immigrants.

## Introduction

Globerman (1998:31), in his study on the health care utilization patterns of immigrants, concludes that "age is the strongest single determinant of health problems" regardless of immigrant status; in fact, his research suggests that immigrants and the native-born utilize health care resources in similar ways at all stages of the life course including in old age [1]. According to Globerman, a *healthy immigrant effect *(HIE) does not exist with regard to the use of health care services even in later life.

In an attempt to interrogate this finding, Gee, Kobayashi and Prus (2004) use a population health perspective to examine the relationship between length of residence (time since immigration) and health status in mid- to later-life individuals [2]. Such a perspective recognizes that the immigrant, economic, and demographic (e.g., age, gender, ethnicity, charter language ability) characteristics of individuals, rather than "medical care inputs and health behaviours" (Dunn and Dyck, 2000:2) are the most salient predictors of health status over the life course [3]. The findings from this study indicate that there are indeed differences between recent, longer-term and non-immigrants according to age on global measures of health status; specifically, there is evidence of a HIE for recent immigrants in midlife (45-64 years), but not for older adults (65+ years).

Further support for the inclusion of age and other markers of inequality like gender and ethnicity as controls in studies on the HIE is found in Newbold's (2005) study on the changing health risk among immigrants [4]. Using longitudinal data from the National Population Health Survey (1994/5-2000/01), he finds that females and young adults (aged 20-34 years) have a lower risk of declining health status relative to males and other age groups, and that Blacks have an increased likelihood of transitioning from a healthy to unhealthy state than other racial groups. Such findings in the Canadian context, a developed country with the highest per capita net immigration rate in the world, provide a foundation on which to further explore the significance of these factors alone and in intersection in studies of immigrant health in other countries with rapidly growing visible minority foreign-born populations like the United States, the United Kingdom, and Australia, and to examine the policy implications of such research in the health care domain.

The current study takes up this challenge in seeking to answer the questions, "Does age, gender, and ethnicity, key markers of social inequality, matter in assessing the health of immigrants? And, if so, in what ways?" By including age as a salient marker of inequality, the study: (1) adds to an HIE literature that has, to date, been largely focused on examinations of the influence(s) of ethnicity and/or gender on immigrant health; and (2), based on its findings, identifies and proposes a policy response(s) to health inequalities among immigrant populations that, despite differences in immigration and health care policies, can be translated cross-nationally to countries, i.e., the US, UK and Australia, where similar results for the HIE have been found (Kennedy, McDonald, and Biddle, 2006) [5].

## Methods

The results of this study are based on data from the public-use microdata file of the 2005 Canadian Community Health Survey (CCHS). The sample consists of 132,221 Canadians aged 12 or older living in private occupied dwellings, with an overall response rate of approximately 85 percent. Adjusted sample weights were used to account for unequal probabilities of selection and non-response in the multistage stratified cluster sampling design employed in the CCHS.

The independent variable, country of birth, is dichotomized as Canadian-and foreign-born. The foreign-born are further classified by length of time in Canada since initial immigration (0-9 years and 10+ years) and cultural/racial origin (non-White and White).

Self-rated health (SRH) is used to measure health status, the dependent variable. SRH indicates a respondent's health status based on his or her own judgment. SRH is a very useful indicator of the overall health and well-being of individuals and populations.

Self-rated health is operationalized in the CCHS as follows: "In general, would you say your health is: excellent, very good, good, fair, or poor?" It was grouped into two categories for purposes of analysis, "poor" (poor or fair) and "good" (good, very good or excellent) health, as has been done in many other studies using self-rated health as an outcome variable. As a measure of health, the reliability and validity of self-rated health have been well-established (Idler & Benyamini, 1997) [6]. SRH provides a valid assessment of overall health (Idler, Russell, & Davis, 1992) [7], and is a strong predictor of mortality (Mossey & Shapiro, 1982; Smith, Shelley & Dennerstein, 1994; van Doorslaer & Gerdtham, 2003), disability (Mansson & Rastam, 2001), functional limitation (Idler & Benyamini, 1997), health-related behaviour (Cott, Gignac, & Badley, 1999; Manderbacka, Lahelma & Martikainen, 1998), and health care utilization (Pinquart, 2001) [7-14]. Banks, Marmot, Oldfield, and Smith (2006) find that self-reported measures of health are almost identical to those with biological measures such as physical and laboratory examinations [15].

Control variables used in the analysis include: age in years (and age square); education (less than a high school diploma vs. high school diploma or higher), household income (before taxes); number of years smoked (for current daily smokers only; all others are coded as 0); and Body Mass Index (under- or overweight, where BMI is < 18.50 or > 24.99, vs. other).

Education and income have a large number of missing cases compared to the other variables. A linear regression substitution approach was used to handle missing income data. A regression was used to predict what a missing score "should be" on the basis of demographic (age and sex) and other economic (labour force status) variables, The missing income data were then replaced with these predicted scores. A dummy variable was constructed to indicate missing vs. non-missing education data. Additional file 1 table S1 provides information on all variables used in the study.

Logistic regression is used to model health for adults (45+ years) across immigrant/visible minority groups. Three models are progressively developed. Model 1 shows the main effects of immigrant status (new immigrant, long-term immigrant, and Canadian-born) on health. Model 2 further distinguishes between visible minority statuses: white and non-white immigrants. The inclusion of this variable allows us to examine if any immigrant status disparities in health observed in Model 1 are due to visible minority status; that is, to examine if a healthy immigrant effect actually reflects visible minority differences in health. Model 3 repeats the analysis in Model 2 with controls for age, age square, education, income, BMI, and years of smoking. Controlling for these factors allows us to see their impact on immigrant/visible minority differences in health. The Pearson chi-square goodness-of-fit test, χ^2^, is reported to assessment of the overall fit of the model.

Additional file 2 table S2 reports the findings of the regression analysis. An odds ratio less than one indicates that the group is less likely to report poor health relative to Canadian born (the reference category). An odds ratio greater than one indicates that the group is more likely to report poor health compared to Canadian born. The regression analysis was done separately for males and females within two adult age groups: 45-64 years and 65+.

## Results

### Distribution of Demographic, Economic, Lifestyle, and Health Variables by Age and Sex

Additional file 1 table S1 reveals a steep age gradient in health. Rates of poor health nearly double from mid- to old-age for males and females. The results, however, do not show a gender difference in health. Males and females report similar rates of poor health in middle- and -old age.

The data also show that the ethnic composition of the population does not change from middle- to old-age for either males or females, though non-whites make-up a much larger share of the male population. Non-whites make-up 13.6 and 12.8 percent of the male population at ages 45-64 and 65+. The comparable figures are 8.6 and 9.0 percent for females. On the other hand, the proportion of newer immigrants in the population does vary by age. About three percent of middle-aged males and females are recent immigrants compared to just one percent of older males and females.

Differences in education and income by age are also revealed in the data. On average older persons, especially women, have lower incomes and levels of education compared to middle-aged persons. Finally, males, particularly in middle-age, are more likely to report unacceptable weight and cigarette consumption compared to females.

### Effects of Immigrant/Visible Minority Status on Health in Midlife - 45-64 Years of Age

Turning to Additional file 2 table S2, the data show strong support for a *healthy immigrant effect *among midlife males. Recent male immigrants, those that have been in Canada for less than 10 years, are significantly less likely to report fair/poor health compared to Canadian-born males (O.R. = 0.26, p < .01). See Model 1 in Additional file 2 table S2.

Further examination of the data suggests that there is a gradient of deterioration in health with time since immigration. That is, there is a convergence in health differences between immigrant and Canadian-born men. There is no significant difference in self-rated health between longer-term immigrant men, those that have been in Canada for 10 or more years, and Canadian-born men (O.R. = 1.04, p > .10).

Looking at Model 2 in Additional file 2 table S2, we see the healthy immigrant effect among recent male immigrants is largely dependent on visible minority status. The health advantage of recent immigrants is particularly strong for non-Whites, whose odds of reporting poor/fair health are 85 percent lower relative to Canadian born persons (O.R. = 0.15, p < .01). By comparison, the odds for recent White immigrants are only 35 percent lower (O.R. = 0.65, p < .10). Interestingly, the inclusion of controls has limited impact on these findings (Model 3). The health advantage of recent, non-white immigrant men is not accounted for by differences in age, economic status, or health behavioral factors. These findings seem to contradict the argument that a healthier immigrant population stems from advantages in demographic, economic, and lifestyle factors, at least as they are measured in the current study. Instead, our findings show that this HIE is mainly attributable to ethnicity; that is, to the exceptionally good health of non-Whites.

The data for middle-aged females, on the other hand, are not consistent with a HIE. Foreign-born females, regardless of how long they have been in Canada, are significantly more likely to report fair/poor health compared to Canadian-born females (Model 1, Additional file 2 table S2). Further, they tend to be disadvantaged in health regardless of visible minority status (Model 2) and age, economic, or health behavioral factors (Model 3).

### Effects of Immigrant/Visible Minority Status on Health in Later Life - 65+ Years of Age

The last three columns of Additional file 2 table S2 show the relationship between immigrant/visible minority status and health for persons 65 years of age and older. These results differ in a few important ways from those reported among persons 45-64. First, recent male immigrants, namely non-Whites, are significantly more likely to report poor/fair health compared to the Canadian-born (O.R. = 2.36, p < .01) (Model 2). This is in opposition to the findings reported for their younger (age 45-64) counterparts. These results are relatively unaffected by controls (Model 3).

Second, unlike their male counterparts, non-White elderly female immigrants who have been in Canada for less than 10 years are actually less likely to rate their health negatively compared to Canadian-born females (O.R. = 0.46, p < .05) (Model 2). Their health does, however, become more comparable to that of older Canadian-born females when the data are adjusted for demographic, economic, and lifestyle differences (O.R. = 0.57, p > .10).

## Conclusions

The findings from this study on whether markers of social inequality matter in assessing the health of immigrants indicate that the answer to this question is yes. The more important inequity question to consider, however, is how do they matter? In responding to this inquiry, we turn to a discussion on how the healthy immigrant effect plays out vis-a-vis these markers. The *healthy immigrant effect *applies to midlife males. Specifically, recent - those who immigrated less than 10 years ago - immigrant men between the ages of 45 and 64 years have better self-rated health compared to the Canadian-born. And, upon further examination, the results suggest that there is a convergence in health differences between foreign- and Canadian-born men in midlife. Interestingly, the health advantage of recent immigrants is especially strong for visible minorities, and is not accounted for by differences in age, economic status, or health behaviors between the immigrant/visible minority groups. This contradicts the argument that a healthier immigrant population can be attributed to advantages arising from such factors. In contrast, the findings are not consistent with a HIE among midlife women. Foreign-born women ages 45-64, regardless of ethnicity, years since immigration, or controls for demographic, economic, and lifestyle factors, are disadvantaged in health compared to Canadian-born women. This disadvantage may reflect differences in the health status of immigrants who enter Canada under different classes; that is, the poorer health status of midlife women, particularly visible minority women (i.e., South Asian, Chinese), may be partially attributed to the fact that they are more likely to have entered the country as family-sponsored immigrants than men (who enter under independent, professional or skilled worker or business classifications). Family-sponsored immigrants come in as "dependents" and this may indicate or suggest that they may not be as "physically" resilient as their "independent" (male) sponsors. This initial vulnerability may endure over the course of their mid-life years and well into later life, especially if access to health care services and programs is (and continues to remain) an issue due to larger cultural (i.e., incongruence in health beliefs, family versus individual decision-making processes) and/or social structural (i.e., lack of appropriate and/or adequate policies and programs) issues.

A different picture emerges in old age. For older men, recent immigrants, particularly visible minorities, are more likely to be disadvantaged with regard to self-reported health even after controlling for key factors. On the other hand, recent visible minority immigrant women in the latter stages of the life course fare much better on self-reported health. This advantage, however, disappears when the data are adjusted for other differences.

Based on these findings, a discussion of the implications for health care policy and program planning for immigrant men and women in mid- to late adulthood - individuals that make up over one-half of the foreign-born adult population in Canada and increasingly larger proportions of the populations in the US, UK and Australia - is warranted. In particular, the findings underscore the necessity for policymakers in such immigrant-receiving countries to address the differential health care needs of immigrant adults by gender and age group. Recent immigrant visible minority men in midlife and, to a lesser extent, their later life female counterparts may have fewer needs for services and programs in the early years of their residency, while certain new immigrant sub-groups, namely older men and midlife women of color may actually have increased needs for services due to poor health status at migration. It should be underscored that this increased need is likely to continue for these women as they age, especially if they experience social isolation (due to geographic, language and/or cultural barriers) and/or under- or unemployment for long periods of time (due to discrimination, etc.). In response to this reality, it is important that policies and programs be developed at both the national and province/state levels, particularly in geographic areas (i.e., around urban centers) in which the majority of new immigrants often choose to reside, that: (a) target midlife immigrant and certain sub-groups of older immigrant women as they age over time; and (b) respond to the needs of an older immigrant male population from the outset.

Specific policy recommendations include the need to actively incorporate a health promotion framework in public health policy. To this end, policymakers must move beyond the funding of large-scale health promotion programs that mainly target children and adolescents in schools, i.e., *Participaction*, to developing programs that are relevant and accessible to the increasing number of adult Canadians, a significant proportion of whom are foreign-born, who are aging with or at significant risk of developing chronic disease. Complementing a dual focus on prevention and treatment, such a policy agenda calls attention to a broad range of social determinants of health and illness that differentially affect the health care utilization patterns and health status of immigrant men and women at various stages of the adult life course.

Finally, despite differences in demographic composition and policy frameworks in the immigration and health care domains of the UK, US, Canada and Australia, Kennedy, McDonald and Biddle (2006) find that there is "evidence of strong positive selection effects for immigrants from all regions of origin in terms of education" in their study of the HIE [5]. This finding provides some empirical support for the cross-national application of the current study's findings and subsequent policy discussion to other large immigrant-receiving countries.

### a. Limitations of the Study

Although the Canadian Community Health Survey (CCHS) provides information on the health status and health care needs of adult Canadians, there are a number of limitations in using these data for this study. First, despite the fact that its data allows for an examination of health status and health care utilization among immigrants, the survey does not collect information on immigrant status or on the reasons for immigrants' entry into Canada. Hence, a more detailed analysis of immigrant men and women's health is not possible; that is, important variations in health status among naturalized citizens, landed immigrants, refugees, and non-permanent men and women cannot be examined in this study.

Second, while CCHS respondents who could not understand English or French were interviewed in their own language, linguistic (as well as cultural) barriers faced by new immigrants may still prevent them from consulting health-care professionals, resulting in an under-diagnosis of health problems (Laroche, 2000) [16]. Cultural factors like adherence to traditional values and beliefs may also influence an individual's willingness to report health problems (Ali 2002; Kopec, Williams, To, and Austin, 2001), since there may be differences in their fundamental conceptualizations of health and illness (Saldov, 1991) [17-19]. Subjective measures of health, like self-rated health, may be affected by differences in "thresholds" used by individuals or groups in assessing their health status (Franks, Gold, & Fiscella, 2003; Schnittker, 2005; Simon, De Boer, Joung, Bosma, & Mackenbach, 2005) [20-22]. It is not unreasonable to assume that the meaning, interpretation, and reporting of self-rated may change across age groups, cultures, and ethnicities.

The extent to which cultural and language differences in the Canadian population influence the interpretation and reporting of health problems is not well known. The magnitude of the differences in men's and women's health status between immigrant and Canadian-born populations reported here, however, make it unlikely that cultural factors exclusively may explain these results.

Third, despite the evidence provided in this study, longitudinal data are needed to verify a true convergence in health status between immigrants and native-born persons over time. It is not possible with the cross-sectional data used here to rule out a cohort effect, whereby differences in men's and women's health among immigrant groups are partly due to the country of birth of immigrants. Longer-term immigrants are more likely to be from Europe and recent immigrants from non-European regions, and both regions vary in terms of general population health - today's immigrants may make-up a healthier cohort than cohorts who immigrated earlier - and in the type and quality of health care systems. Health requirements for entry into Canada (as well as the US, UK and Australia) have also changed, i.e., become more stringent, over time (Perez, 2002) [23]. It should also be noted that, as we are unable to acquire standardized health status and utilization data pre-immigration from all source countries, the validity of findings on the HIE may be called into question even if longitudinal data were collected post-immigration.

Finally, the CCHS data used for this study are limited in two ways. First, age is defined in five-year groups (e.g., 45-49 years) as opposed to respondents' actual age. Subsequently, some of the key variations between immigrants and non-immigrants may be due to small differences in the average age of respondents within each of their age cohort groups. Second, these data do not allow for the consideration of a key variable such as ethnicity (i.e., country of birth) as both a control and independent variable in the current analyses.

## Competing interests

The authors declare that they have no competing interests.

## Authors' contributions

KK did the literature review and wrote the introduction. SP wrote the methods section and carried out the data analysis. Both KK and SP wrote the Results and Conclusions sections, and read and approved the final manuscript.

## Supplementary Material

Additional file 1 **Additional file 1 table S1 Sample Characteristics by Age and Gender**. Click here for file

Additional file 2 **Additional file 2 table S2 Odds Ratios for Immigrant/Visible Minority Status Differences in Poor/Fair Self-Reported Health, by Age and Gender**. Click here for file
