# Supplementary material for: Examining the gender, ethnicity, and age dimensions of the healthy immigrant effect: Factors in the development of equitable health policy
Source: Int J Equity Health. 2012 Feb 16;11:8. doi: 10.1186/1475-9276-11-8 (PMC3305534; doi:10.1186/1475-9276-11-8)
Supplement: Additional file 1 — table S1 Sample Characteristics by Age and Gender. [file 1475-9276-11-8-S1.PDF]

Additional file 1 table S1 Sample Characteristics by Age and Gender

	45-64	65+
**MALE**		
Poor/Fair Health, *%*	13.4	26.3
Immigrant Status, *%*		
FB, <10 yrs	3.4	1.0
FB, 10+ yrs	20.4	27.8
CB	76.2	71.2
Visible Minority (Non-White), *%*	13.6	12.8
Age (in years), *M* (*SD*)	53.6 (5.4)	73.2 (5.5)
<High School, *%*	15.7	36.7
Income (in dollars), *M* (*SD*)	73,592 (36,520)	44,226 (27,867)
Unacceptable Weight, *%*	66.4	60.5
Years Smoke, *M* (*SD*)	6.9 (14.3)	5.0 (15.7)
**n**	**20,090**	**8,519**
**FEMALE**		
Poor/Fair Health, *%*	14.0	26.4
Immigrant Status, *%*		
FB, <10 yrs	3.1	1.2
FB, 10+ yrs	19.7	23.9
CB	77.2	74.9
Visible Minority (Non-White), *%*	8.6	9.0
Age (in years), *M* (*SD*)	53.5 (5.4)	74.1 (5.8)
<High School, *%*	15.4	43.2
Income (in dollars), *M* (*SD*)	65,586 (36,549)	36,003 (25,645)
Unacceptable Weight, *%*	52.5	53.8
Years Smoke, *M* (*SD*)	5.9 (13.2)	3.8 (13.5)
**n**	**20,550**	**10,627**

FB, <10 yrs: foreign-born, less than 10 years in Canada
FB, 10+ yrs: foreign-born, 10 or more years in Canada
CB: Canadian-born
