# Supplementary material for: Examining the gender, ethnicity, and age dimensions of the healthy immigrant effect: Factors in the development of equitable health policy
Source: Int J Equity Health. 2012 Feb 16;11:8. doi: 10.1186/1475-9276-11-8 (PMC3305534; doi:10.1186/1475-9276-11-8)
Supplement: Additional file 2 — table S2 Odds Ratios for Immigrant/Visible Minority Status Differences in Poor/Fair Self-Reported Health, by Age and Gender. [file 1475-9276-11-8-S2.PDF]

Additional file 2 table S2 Odds Ratios for Immigrant/Visible Minority Status Differences in Poor/Fair Self-Rep
Health, by Age and Gender

	(1)	(2)	(3)	(1)	(2)	(3)
	**45-64**			**65+**		
**MALE**						
**Immigrant/Visible Minority Status**						
FB, <10 yrs	0.26***			2.11***		
White		0.65*	0.74		1.01	1.31
Non-White		0.15***	0.15***		2.36***	
3.03***						
FB, 10+ yrs	1.04			0.96		
White		1.11*	1.20***		0.98	1.09
Non-White		0.97	1.11		0.93	1.17
CB	1.00	1.00	1.00		1.00	1.00
χ^2	71.0***	86.7***	1228.0***	9.8***	11.9**	
485.4***						
**FEMALE**						
**Immigrant/Visible Minority Status**						
FB, <10 yrs	1.46***			0.71		
White		1.75***	2.28***		1.90*	
2.22**						
Non-White		1.36**	1.40**		0.46**	0.57
FB, 10+ yrs	1.37***			1.43***		
White		1.24***	1.34***		1.23***	
1.28***						
Non-White		1.58***	1.96***		2.05***	
2.35***						
CB	1.00	1.00	1.00	1.00	1.00	1.00
χ^2	48.9***	60.0***	1482.6 ***	51.0***	85.2***	
564.0***						

FB, <10 yrs: foreign-born, less than 10 years in Canada (white and non-white)
FB, 10+ yrs: foreign-born, 10 or more years in Canada (white and non-white)
CB: Canadian-born (reference category)

Model 1 (1) shows effect of immigrant status on health
Model 2 (2) shows effect of immigrant, visible minority status on health
Model 3 (3) repeats Model 2 controlling for age (and age square), education, income, BMI, and years of smoking.

*** = p < 0.01, ** = p < 0.05, *= p < 0.10.
